# The Midwives' Bill: A Select Committee

**Published:** 1891-03-28

**Authors:** 


### The Hospital

A Weekly Journal of

Science, Medicine, Nursing, and Philanthropy.

*For Hospitals, Asylums, Infirmaries, Dispensaries, Medical Practitioners, Students, Nurses, and the Charitable Public.*

Vol. IX.—No. 235.] [March 28, 1891.

### The Midwives' Bill: A Select Committee.

A sober discussion of the Midwives' Registration Bill took place at the Hospital for Women last week under the auspices of the Hospitals' Association. Dr. Farquaharson, M.P., presided, and Mrs. Henry Smith, President of the Midwives' Institute, and Dr. Leith Napier, each read a paper. The meeting was open and representative, and at the close the following resolution was passed, "That in view of the important questions involved in the legal registration of midwives, this meeting of the Hospitals' Association requests the Council of the Association to memorialise the Government in favour of a Select Committee with the view of securing the early registration of midwives." A Select Committee appointed for the purpose of completely investigating the subject will be a practical movement in a forward direction.

Class interests always find clamorous defenders; but if classes were keen-witted they would learn to be suspicious of those who undertake to defend their cause. Public movements must have leaders, but the leaders generally partake of the character of the movements. If a particular agitation be mainly impelled by selfish motives, it is very likely indeed that those who place themselves at the head of it will have selfish motives too. It does not follow that their motives will be identical with those which impel the agitation. On the contrary it will usually be found that the motives of such leaders are personal and peculiar to the leaders. In other words, whilst a man may seem to be devoting himself heart and soul to a cause which he leads, he may be really using the cause for his own personal ends. Of course, this is by no means always the case, neither is it more frequently, or less frequently the case, in the medical profession than among other bodies of men. What ought to be impressed upon readers is that medical men will act from precisely the same motives as all other classes of the people; and that with all people self interest is a motive which is seldom in the background.

It is not difficult to sympathise with those medical men who think they see a portion of their livelihood endangered by the registration of midwives. A very large proportion of practising doctors find it almost impossible to make both ends meet. The public does not know, consulting physicians and surgeons do not know, and the more prosperous among the general practitioners do not know how stern is the struggle, and how wearing and exhausting are the labours of the average doctor whose work lies among the lower middle and the poorer classes of the population. If such doctors could work a definite number of hours every day—even if those hours were very long—and could then be sure of one or two hours' rest in the evening and of spending the whole of the night in uninterrupted and refreshing sleep, they might carry on their exhausting labours to old age like other workers. But the night work, the terrible night work, added to the interminable toils and worries of the day, makes of the lives of many doctors what can only be described as stern and bitter tragedies. If medical men in these circumstances hold fast with tenacious grasp even to a very few pounds of annual income, who shall wonder at it? They are already called upon in the exercise of their daily avocation to do a very large amount of purely gratuitous work. Many of them never expect to get more than sixty or seventy per cent. of what they actually earn. In certain neighbourhoods hospitals compete with them on the one hand, charging no fees at all; on the other, are unscrupulous practitioners, who hesitate at no tricks, however disgraceful, if they can but bring a few miserable shillings to their own till. Doctors, those doctors who bear the brunt of the medical work done for the masses of the people, give more brain, and heart, and bodily energy to the public for an inadequate remuneration than any other class.

Over against these undoubted facts are to be set the distresses, the sufferings, and the dangers to which poor women are exposed, in this civilised and right-thinking country, at the time of child-birth. Whilst we see clearly, and willingly admit, the great merits and sharp straits of the practitioners who work for the masses, we must not permit ourselves to overlook the equal moral worth and still sharper straits of those poor working-men's wives who do their duty in their families under even a greater pressure and a severer struggle than that which falls to the lot of the doctor. The working man's wife who has a large family may be no heroine, but she is certainly often a martyr. The manifold nature of her toils, the variety and urgency of her anxieties, the extreme inadequacy of her resources, and the hopeless outlook of the near future, constitute an environment which any thoughtful man might contemplate for his own daughter with terror and dread. The scales have to be held between the hard-driven doctor on the one hand, and the more sorely-pressed wife and mother who lives and brings forth children in poverty on the other.

It is clear, then, that the subject is one which ought not to be left in the hands of impulsive rhetoricians and unstatesmanlike partisans. If ever there was a case which demanded a full disclosure of all the facts, a careful weighing of all the evidence, and a sympathetic and discriminating judgment which will give due consideration to the just claims of both parties, it is the question of the education and registration of midwives. The friends of registration have had their say on the one side, and the opponents have had theirs on the other. But the speakers have been too numerous, and their speech too noisy for those who will have to give the ultimate decision to be able to form a correct judgment. What are now wanted are witnesses who are familiar with the facts; and to them must be given opportunities of speaking without passion and without prejudice. The medical profession consists of educated men. They ought to be able, and they are able, undoubtedly, to look the facts squarely in the face, and to consider them from the standpoint of disinterested spectators.

The proposed Select Committee will give both to the opponents and to the friends of the Bill an opportunity of pitting their evidence over against each other. Now that it has been suggested it is to be hoped that it will be speedily constituted. The matter cannot rest where it is, and so long as it continues to be agitated, so long will it stir up strife and angry passions among members of the same profession, who ought to be friends. Already the question is assuming the character of a chronic quarrel between opposing factions; and the real point at issue is rapidly being forgotten. Many a bloody war in earlier times has been prolonged long after the original *casus belli* had ceased to be of any practical moment. But such strifes were both stupid and criminal. Men of honest hearts and discriminating minds do not quarrel about shadows: their self-respect, and their regard for what is just and right and seemly, restrain them from such foolish and childish behaviour. The medical profession, unhappily, has the reputation of being very quarrelsome within its own borders. "Doctors' squabbles" have become bye-words and epithets of contempt in the mouths of a robust-minded public. But surely the medical profession is not to be regarded for ever as a feeble imitation of the cockpit!

It is a pity that the poor women who are most concerned cannot come forward and plead their own cause. But their cause must be pleaded! Those who know the facts and who sympathise with the women must have the courage of their opinions. Loud-voiced clamour must be valued at its true worth. It is just as necessary to stand by convictions in matters that do not concern ourselves as it is in circumstances where our own peculiar interests are involved. There is nothing so contemptible as running with the crowd merely because it is the crowd. Man has been endowed with a mental and moral backbone in order that he may stand upright against all conceivable odds in a just cause. It cannot be an unjust cause which proposes to improve the available means of help for poor and distressed women. The cause of the poor indeed is mostly just, since they have not the power to gain any sympathy from the better classes except when it is just. It is a good rule to do as one would be done by.

